# Transient stabbing headache from an acute thalamic hemorrhage

**DOI:** 10.1007/s10194-011-0303-y

**Published:** 2011-02-05

**Authors:** Matthew S. Robbins

**Affiliations:** Montefiore Headache Center, The Saul R. Korey Department of Neurology, Albert Einstein College of Medicine, 1575 Blondell Avenue, Suite 225, Bronx, NY 10461 USA

**Keywords:** Stabbing headache, Secondary headache, Thalamus, Intracranial hemorrhage

## Abstract

Stabbing headache can be encountered in both primary and secondary forms, but has been infrequently reported among patients with stroke, and is not known to be associated with a small well-circumscribed brain lesion. A 95-year-old woman taking warfarin presented with the sudden onset of stabbing headache strictly in the right frontal and supraorbital regions, along with gait imbalance and dysarthria. Neuroimaging revealed a small left thalamic hematoma. This association of an acute thalamic lesion with stabbing headache in the contralateral trigeminal distribution is discussed, along with a brief review of stabbing headache occurring in cerebrovascular disease.

## Introduction

Primary stabbing headache is a short duration headache disorder that is characterized by brief paroxysms of sharp head pain often located in the first division of the trigeminal nerve, occurring at an irregular frequency, lacking any accompanying symptoms, and not attributable to any underlying cause [1]. Stabbing headache has also been reported to occur as a symptom of, or in association with, a variety of underlying secondary disorders, including giant cell arteritis [2, 3], pituitary tumors [4], meningiomas [5], ocular pathology [6], ischemic stroke [6, 7], cavernous hemangioma of the frontal bone [8], and varicella zoster meningoencephalitis [9]. Stabbing headache has not been reported to manifest as a direct consequence of a small well-circumscribed acute brain lesion, and its pathophysiology remains elusive. Herein, a lesional cause of stabbing headache, a small acute thalamic hematoma, is presented.

## Case report

A 95-year-old right-handed Caucasian woman presented to our emergency department with the sudden onset of a constellation of spontaneous neurological symptoms. She first noted repetitive, sharp, 1–2 s paroxysms of pain in strictly the right frontal and supraorbital region, occurring dozens of times per hour but at irregular and unpredictable frequencies. The pain was severe, stabbing, and unwavering in its location. Her pain was unaccompanied by photophobia, phonophobia, cranial autonomic symptoms, or any visual changes. She did not identify any triggers and tactile stimulation of any area of the head or face did not provoke the pain. No analgesics were taken by the patient at home or in the hospital. These attacks continued until she finally fell asleep in the hospital 12 h later, and upon awakening 6 h later, did not recur.

Minutes after the head pain onset, she noted mildly slurred speech, which also resolved by the following morning, and upon attempting to ambulate she felt very imbalanced, which persisted for several days.

Her past medical history included hypertension, hyperthyroidism, gastroesophageal reflux, osteoarthritis, and a left intertrochanteric femoral fracture sustained after a fall, which was surgically repaired 4 months previously. Postoperatively she developed cholangitis from choledocholithiasis, an acute pulmonary embolism, and paroxysmal atrial fibrillation, for which she was treated with intravenous antibiotics and placed on long-term anticoagulation.

She had no history of any primary headache disorder, denied ever experiencing any focal neurological symptoms. Her medications included warfarin, fosinopril, omeprazole, metoprolol, digoxin, and methimazole. She was not a smoker and did not consume alcohol. She lived with her husband and was a retired school teacher. On review of systems, for several months she had ongoing right upper quadrant pain and occasional nausea that had been attributed to choledocholithiasis. She denied jaw claudication, episodes of visual loss, myalgias, or neck pain.

Her general medical examination revealed a well-appearing woman with normal vital signs, and only mild right upper quadrant tenderness. On neurological examination, her mental status and cranial nerves were normal, including fundoscopy. She had no ptosis, facial sensory deficits, and was no longer dysarthric. Her motor examination was normal aside from an upward drift of the right arm. Deep tendon reflexes were 1+ in the arms, trace at the knees, and she lacked ankle jerks. She had bilateral Babinski signs. Sensation was intact to all modalities, and her Romberg sign was negative. She had no tremor, dysmetria, or dysdiadochokinesia. Her gait was somewhat cautious, slightly wide based, and with increased sway.

**Fig. 1:**
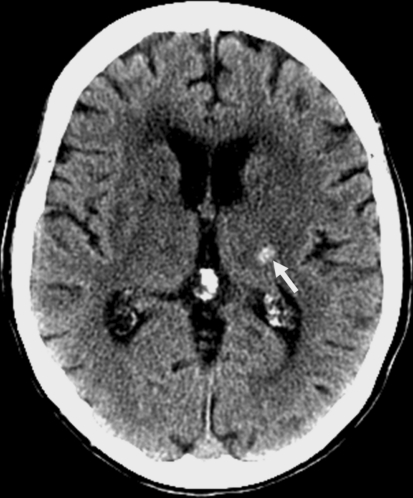
Axial noncontrast computed tomography of the brain revealed an acute, rounded hyperdense lesion in the region of the left thalamus and likely abutting the posterior limb of the internal capsule (*white arrow*), corresponding to a small acute hemorrhage

Laboratory data revealed normal serum chemistry, hematocrit, and platelets. She had a white blood cell count of 12,000/μl, a total bilirubin of 2.3 mg/dL, a direct bilirubin of 1.2 mg/dL, an alkaline phosphatase of 560 U/L, a SGOT of 341 U/L, and a SGPT of 292 U/L. Her international normalized ratio was 3.2. An electrocardiogram revealed normal sinus rhythm. Noncontrast computed tomography of the head revealed a rounded hyperdense lesion in the left lateral thalamus abutting the posterior limb of the internal capsule, consistent with an acute small hemorrhage, along with multiple subcortical lacunes and white matter ischemic changes (Fig. 1). The hematoma volume was estimated to be 0.12 cm^3^ using the ABC/2 method [10].

The patient was administered fresh frozen plasma and vitamin K to reverse her coagulopathy and ultimately had an endoscopic retrograde cholangiopancreatography, where numerous gallstones were removed. Serial neuroimaging revealed the hematoma was slightly decreasing in size. The patient was ultimately discharged to a short-term rehabilitation facility for her imbalance and deconditioning.

## Discussion

This patient experienced stabbing headache as the first symptom of a small thalamic hemorrhage. The correspondence of the acuity of the clinical syndrome with the radiographic demonstration of an acute hematoma strongly supports its causality, although the lack of magnetic resonance imaging makes the exclusion of other acute lesions slightly less definitive.

Stabbing headache has been reported as a symptom of many underlying disorders. A fixed site of stabbing headache may signify an underlying symptomatic cause of the disorder, [1] as was the case with this patient.

Although a stabbing pain character of headache is not uncommon in association with acute cerebrovascular disease, the diagnosis of stabbing headache has not been well described in this population. In a large prospective series of over 2,000 patients with acute ischemic stroke where headache details were captured at stroke onset, 20% of the patients described their pain character as stabbing, although the diagnosis of stabbing headache was not mentioned [11]. Pareja and colleagues mentioned the onset of stabbing headache in a patient with an ischemic stroke to the parietal lobe, but did not describe its laterality and relationship to the site of the stabbing head pain [6]. Piovesan and colleagues reported the occurrence of stabbing headache in a delayed fashion after an ischemic stroke in three patients who all likely sustained acute middle cerebral artery territory infarctions [7].

Regarding hemorrhagic stroke, in a large series of 90 intracranial hemorrhage survivors, six patients experienced stabbing headache de novo after intracranial hemorrhage, only one of whom had no premorbid headache history [12]. The pain distribution in relationship to the hematoma location was not described. Acute thalamic hematomas in particular can lead to headache over half the time, but similar to other deep structures do not lead to headache nearly as often as hemorrhage in the posterior fossa or lobar structures [13].

In this patient, stabbing headache resulted from an acute parenchymal brain lesion in the thalamus, a location vital to the processing of pain. More specifically, the location of the hematoma may have encompassed the ventral posteromedial nucleus of the thalamus, a relay center for sensory input from the trigeminal system. The association of the pain strictly occurring in the right trigeminal distribution with a lesion in the corresponding left thalamus is intriguing.

The pathophysiology of stabbing headache is not known. Some authors have proposed the disorder results from spontaneous activation of peripheral nerve branches in the trigeminal or upper cervical distribution [14, 15]. Another theory is that patients with stabbing headache have segmental and fleeting disinhibition of central pain pathways [14, 15]. Perhaps in cerebrovascular disease, such disinhibition occurs in an acute fashion in those patients with contralateral pain to the site of infarction or hemorrhage. In stroke patients with ipsilateral stabbing headache, the head pain may reflect referred pain from blood vessel or meningeal involvement.

This report demonstrates an association of an acute thalamic lesion with stabbing headache occurring in the contralateral trigeminal distribution. Particularly in an elderly patient or individual taking anticoagulants, a small thalamic hemorrhage should be considered in the differential diagnosis of new-onset stabbing headache, even when fleeting.
